# Ovariotomy by Enucleation, without Clamp, Ligature or Cautery, and without Hemorrhage

**Published:** 1871-08

**Authors:** 


### Ovariotomy by Enucleation, without Clamp Ligature or Cautery, and without Hemorrhage.

We are much gratified to see that not only the profession in our own country, but also in Europe, are following our suggestion in the treatment of the pedicle in ovariotomy, and that our observations as to the feasibility and safety of the procedure are being sustained by distinguished operators abroad. Our first case has been published about three years; since that time our own obsevations, and the experiments of our friends fully confirm our first impression, founded upon a single case. A proposition so new, and at first thought, so hazardous, could not be rapidly accepted. Every surgeon who has operated frequently, has seen the large throbbing arteries at the base of ovarian pedicle, and they can scarcely believe that tracing up the vessels with their loose cellular surroundings they will so soon arrive at the final termination, where the vessels enter the cyst, (if at all), only of capillary size. Richard H. Meade, Esq., Consulting Surgeon to the Bradford Infi mary, England, thus speaks upon the subject.—

"The patient being under the influence of chloroform, I made an incision about four inches long in the lower part of the lmea alba; carefully opened the peritoneum; and evacuated nearly two gallons of ascitic fluid. On enlarging the opening in the peritoneum to the same extent as the external wound, the ovarian tumor at once came into view. I now directed an assistant to compress the abdominal walls with his hands, one placed on each side, so as to compress the edges of the wound backwards; while I endeavored with my hands to draw the tumor partially through the opening. In doing this, the walls of several of the small cysts of which the tumor was principally composed, (being very thin) were ruptured by the pressure of my fingers; and a considerable quantity of thick brown fluid, like dark-colored linseed tea, escaped. The edges of the wound were so well compressed that none of this ovarian fluid was allowed to enter the peritoneal cavity. The tumor was now found to be firmly adherent to the free extremity of the great omentum; these adhesions were carefully and slowly torn through, and the whole mass was then easily drawn through the wound. It was now found to contain a good deal of heavy solid matter ; and, on turning it over to examine its attachments the pedicle, which was small and thin, to my dismay and annoyance at the time, gave way, and the tumor tore itself loose from its connexions. Fearing hemorrhage, I kept hold of the remains of the pedicle, but very little bleeding followed ; and I could find no vessel requiring ligature.

"It is difficult to account for the large quantity of ascitic fluid which was met with, unless there were some secondary deposits of cancerous matter on the peritoneum; but the history of the case would hardly lead to that supposition. The presence of a considerable amount of serous effusion in the peritoneal cavity has, however, one advantage in cases of ovariotomy; it seems to render the membrane less liable to take on acute inflammation, its delicate secreting surface having undergone some change, and after the removal of the ovarian disease it does not seem to be resecreted.

"In some of the medical journals a case of ovariotomy has been reported (extracted from an American periodical), in which Dr. Julius F. Miner removed a very large ovarian tumor by enucleation, without either clamp, ligature, or cautery, and without hemorrhage. When I commencod the above operation I had no idea of imitating his proceeding, but when the tumor (to my horror at the time) enucleated itself, and there appeared to be no bleeding from the pedicle, I determined to follow his example, and leave the torn surface unsecured ; thinking that the risk from hemorrhage was less than that from inflammation from the presence of a foreign body in the peritoneal cavity. My case turned out successfully, and I think I should venture to repeat the proceeding in some special cases; for instance, where the pedicle does not appear to be vascular, when the attempt might be made to tear the tumor gently from its connexions (in the same way as adhesions are generally separated) ; but a firm hold should be kept of the pedicle, so that it might easily be secured in case of bleeding.—*British Medical Journal.—Braithwaite's Retrospect, for July*, 1871.

